# Elastic Fibers in the Intervertebral Disc: From Form to Function and toward Regeneration

**DOI:** 10.3390/ijms23168931

**Published:** 2022-08-11

**Authors:** Divya Cyril, Amelia Giugni, Saie Sunil Bangar, Melika Mirzaeipoueinak, Dipika Shrivastav, Mirit Sharabi, Joanne L. Tipper, Javad Tavakoli

**Affiliations:** 1 Centre for Health Technologies, School of Biomedical Engineering, Faculty of Engineering and Information Technology, University of Technology Sydney, Sydney, NSW 2007, Australia; 2 Faculty of Science, University of Technology Sydney, Sydney, NSW 2007, Australia; 3 Department of Mechanical Engineering and Mechatronics, Ariel University, Ariel 407000, Israel

**Keywords:** elastic fibers, intervertebral disc, elastic fibers structure, elastic fibers function, elastic fibers development and aging, tissue engineering, computational models

## Abstract

Despite extensive efforts over the past 40 years, there is still a significant gap in knowledge of the characteristics of elastic fibers in the intervertebral disc (IVD). More studies are required to clarify the potential contribution of elastic fibers to the IVD (healthy and diseased) function and recommend critical areas for future investigations. On the other hand, current IVD in-vitro models are not true reflections of the complex biological IVD tissue and the role of elastic fibers has often been ignored in developing relevant tissue-engineered scaffolds and realistic computational models. This has affected the progress of IVD studies (tissue engineering solutions, biomechanics, fundamental biology) and translation into clinical practice. Motivated by the current gap, the current review paper presents a comprehensive study (from the early 1980s to 2022) that explores the current understanding of structural (multi-scale hierarchy), biological (development and aging, elastin content, and cell-fiber interaction), and biomechanical properties of the IVD elastic fibers, and provides new insights into future investigations in this domain.

## 1. Introduction

**Figure 1:**
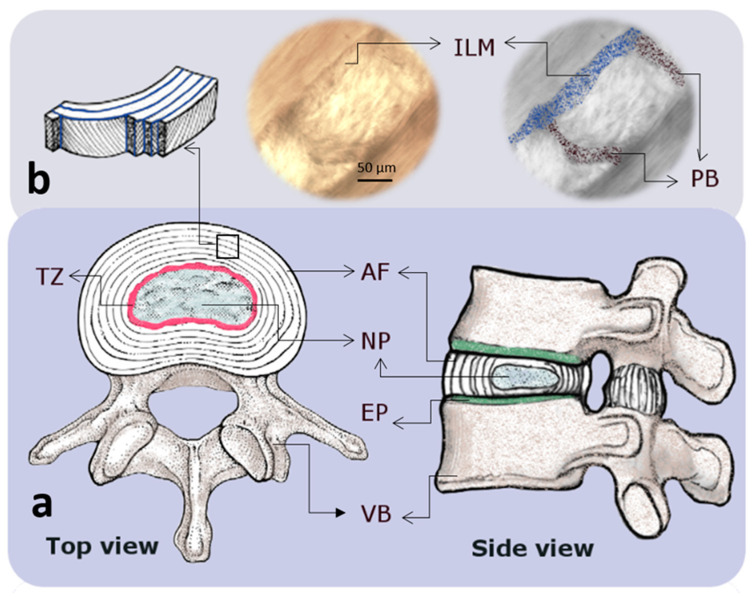
Schematic drawings indicating the macro and microstructure of an intervertebral disc (IVD). (**a**) Top and side views of an intervertebral disc showing the annulus fibrosus (AF) and nucleus pulposus (NP) regions that are confined by endplates (EP) and vertebral bodies (VB). The EP and the region between the AF and NP, known as the transition zone (TZ), are represented by green and pink colors, respectively. (**b**) At the microscale, the AF consists of lamellae (layers) which are made of highly packed collagen fibers that are connected by a network of elastic fibers with a high concentration at the interlamellar matrix (ILM; blue) and partition boundaries (PB; black) [11].

The intervertebral disc (IVD) is a three-component connective tissue that consists of a central gel-like nucleus pulposus (NP) which is surrounded by concentric annulus fibrosus (AF) layers, peripherally, and two cartilaginous endplates that interface with the vertebral bodies (Figure 1a) [1]. The AF is a multi-layer (lamellar) structure, packed with parallel bundles of collagen fibers (mainly type I) that are structured at alternating angles (±30°, with respect to the axial axis) in adjacent layers [2]. Adjacent AF lamellae are separated by a region with an average thickness of less than 30 µm containing a high density of elastic fibers, called the inter-lamellar matrix (ILM) [3]. In addition, each lamella is segmented by a dense structure of elastic fibers, a region recognized as a partition boundary (PB, also known as translemaller fibers), which may divide the entire lamella (connecting adjacent ILMs) or appear as partial dividers (Figure 1b) [4,5]. The AF is attached to the superior and inferior endplates, creating a reinforced structure around the NP. The NP, rich in aggrecan, is a highly hydrated component that contains a delicate meshwork of collagen (type II) organized around the NP cells [6]. A transient gradient, via the transition zone (TZ) that is located at the interface of the AF and NP, from collagen type II to collagen type I is observed in the NP towards the peripheral AF [7,8]. While collagen types I and II are the most abundant components of the IVD’s extracellular matrix (ECM), other minor collagen types (XIV, XII, XI, IX, VI, V, and III) are also found in the healthy and mature IVD [9]. Apart from collagen, different biomolecules such as hyaluronan, glycosaminoglycans, proteoglycans, biglycan, decorin, fibromodulin, keratocan, lumican, perlecan, elastin, lipid, glycoprotein, and catabolic enzymes can be found in the ECM of the IVD [10]. The ECM biomolecules such as collagens and proteoglycans have been the focus of different biomechanical, biochemical, and histological studies; however, the detailed structure–function relationships of elastic fibers in the IVD have been rarely explored until recently. Due to the lack of inclusive descriptions of the architectural design and function of elastic fibers in the IVD, their contribution to healthy IVD function and their potential roles in the progression of IVD degeneration are not very well known.

IVD degeneration is characterized by progressive mechanical and biological mechanisms, leading to irreversible structural failure that impairs IVD function. There is a subtle relationship between the biological and mechanical pathways during progression to IVD degeneration, and their interconnection amplifies the process [12]. The IVD microstructural changes alter the local micromechanical environment that develops cell-mediated responses to initiate or accelerate biological retorts. On the other hand, inadequate metabolite transport, dehydration, and pathogens can disturb IVD cells’ physiological behavior, leading to microstructural changes that consequently alter the mechanical properties of the IVD. This may apply a higher load to the IVD components, cause local stress concentrations, develop tissue clefts, and increase the risk of endplate fracture or IVD failure. The role that different ECM constituents such as collagen and proteoglycans play during the IVD degeneration process has been extensively studied [13,14,15,16,17,18,19]; however, the effect of elastic fibers on the degeneration process and whether they stimulate the biological mechanisms are yet to be explored. Over the past decade, an increasing number of studies have been devoted to the IVD elastic fibers, which has enhanced our understanding of their structural organization and function. However, more studies are required to fully describe the structure–function relationship of elastic fibers in the IVD and their biological role. Therefore, this comprehensive review paper aims to investigate the architecture, mechanical function, developmental biology, and biomimetics of elastic fibers in the IVD to identify the gap, clarify the potential contribution of elastic fibers to the IVD (healthy and diseased) function, and recommend critical areas for future investigations.

## 2. Review Methodology

The current review was prepared by performing a comprehensive search (from the early 1980s to 2022) using PubMed and Web of Science online databases, including papers that were published in peer-reviewed journals. The keywords searched were “intervertebral disc” and/or “elastic fibers”. In addition, the bibliographies of selected papers were employed to find other relevant publications that were not found in the keyword search.

## 3. Multi-Scale Hierarchical Structure of the IVD Elastic Fibers

**Figure 2:**
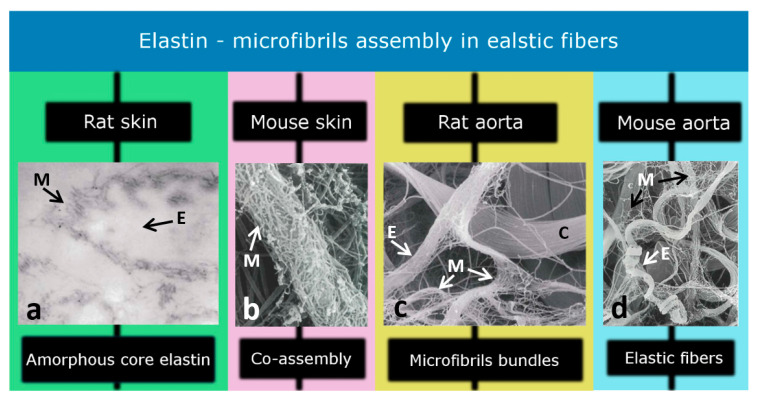
Elastic fibers contain an elastin core (amorphous structure) which is enclosed by microfibrils. Different studies have identified (**a**) the amorphous core region (×125), (**b**) co-assembly of elastin and microfibrils (×55,000), (**c**) microfibril bundles (×11,000), and (**d**) elastic fibers (×9100) in the skin and aorta of the rat and mouse models. M, E, and C represent microfibrils, elastin, and collagen, respectively. [Modified from Ref [20] with permission].

From a structural point of view, revealed by microscopy studies, elastic fibers in soft tissues are composite structures comprising core elastin proteins surrounded by microfibrils (Figure 2a) [20]. TEM observations have revealed the core part of elastic fibers as an amorphous component that can be further purified by hot water (95–100 °C), with the remaining part being biochemically recognized as elastic protein [21]. The microfibrils consist of several glycoproteins, mainly fibrillin and amyloid, and are randomly presented around the elastin core (Figure 2b) [22]. Since microfibrils leave the elastin core to form a continuous bundle of microfibrils, it is thought that they act as anchoring fibers, connecting elastic fibers with the surrounding ECM (Figure 2c). When elastic fibers are exposed to a tensile force, the directionality of microfibrils is changed, with most being directed along with the elastin core. While the role of the elastin core is largely mechanical to drive passive recoil and control the elasticity of the tissue, microfibrils strengthen the elastic fibers [23]. In addition, microfibrils are thought to mediate biological events, including cell signaling, tissue homeostasis, and elastogenesis [24]. Elastic fibers are mostly twisted or straight strands with a thickness of less than 1.5 μm, while microfibrils have a diameter of approximately 0.01 μm (Figure 2d). The morphological feature of the elastic fibers is tissue-specific, which impacts the elasticity of tissue, which is important for the associated biomechanical role. However, elastic fibers often branch and merge to assemble a coarse network in loose connective tissues, while in highly elastic tissues with a high density of elastic content, elastic fibers are more likely to form elastic laminae, microscopically observed as flattened and interwoven elastic sheets [20].

While the structural organization and mechanical role of elastic fibers in different soft tissues, including lungs, tendons, vessels, and skin, have been widely studied, our understanding of their characteristics in the IVD is still limited [25,26,27,28,29,30,31]. Early studies have identified a sparse and irregular distribution of the elastic fibers in human IVD where the elastin content was reported to be less than 2% of the total IVD dry weight [32]. The low quantity of elastin content, compared to collagen, has likely been the main reason for less attention being shown to the role of IVD elastic fibers. Another reason has been the lack of suitable research tools to isolate elastic fibers in-situ, which is important for direct measurement and characterization of their biomechanical properties.

Efforts to identify elastic fibers as a structural component of the IVD date back to 1952. However, the main challenge faced was identifying their presence using light microscopy, which remained unsuccessful [33]. Buckwalter et al. (1976) were the first investigators that observed elastic fibers in human IVD using transmission electron microscopy [34]. Subsequently, the presence of elastic fibers in the borderline between the AF and NP was reported by Sylves et al. in 1977, using samples that were extracted from human prolapsed IVDs [35]. In 1981, Hickey and Hukins extended the observations of Buckwalter by finding elastic fibers in human fetal AF. Elastic fibers in the human fetus were typically immature, consisting of a bundle of microfibrils approximately 20 nm in diameter [36]. Johnson et al. (1982) performed a light microscopy study to identify the arrangement of elastic fibers in the cervical IVD of the adult human and reported that elastic fibers were located only in the AF lamellae and at the interface of the NP and vertebral bodies [37]. They reported a three-dimensional lattice of elastic fibers at the NP–endplate interface with elastic fibers, which penetrated the vertebrae as Sharpey’s fiber. Nevertheless, to ascertain the distribution of elastic fibers, they used dog IVDs (1984) and realized that elastic fibers were present in both the intra- and inter-lamellar regions of the AF [38]. They found a vertical and oblique arrangement for the elastic fibers in the interlamellar region of the outer AF, with a more radially oriented loose network in the inner AF [38]. In 1985, Johnson et al. extended their experiment using human lumbar IVD and found that approximately 10% of the AF extracellular matrix consisted of elastic fibers [39]. Mikawa et al. (1986) reported a very sparse and irregular distribution of the elastic fibers in human IVD where the elastin content, based on the hot alkali method, was less than 2% of the total IVD dry weight [40]. Table 1 shows the outcomes of early studies (1970–2000) to detect elastic fibers and their arrangement in the IVD.

These results suggest that the arrangement and distribution of elastic fibers may change between different species and depend on the IVD level. In addition, the results from these studies confirmed the presence of elastic fibers throughout the IVD (AF, NP, their interface, and even at the endplate junction) and concurrently shattered randomly amongst the collagen fibers. The concept of sparse elastic fibers in the IVD had come to an end in 2002 when Yu et al. reported an abundant and organized distribution of elastic fibers in different regions of bovine IVDs [32].

**Figure 3:**
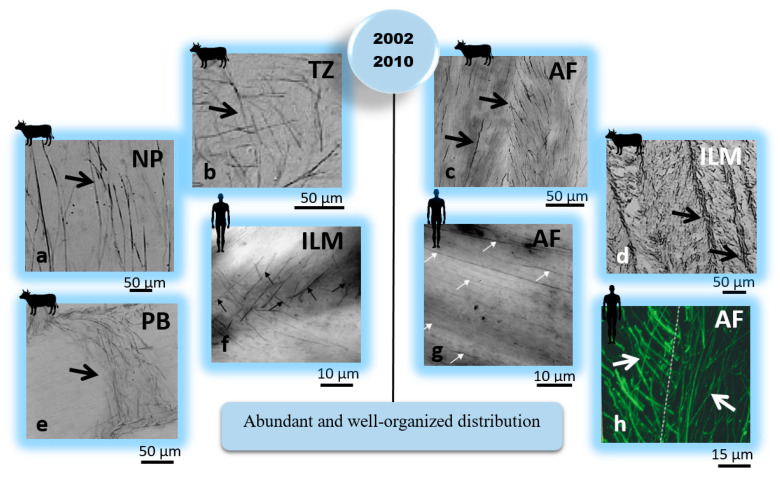
Complementary IVD structural studies (2002–2015) have revealed that the arrangement and distribution of elastic fibers are different across different regions of the IVD. These findings suggested that elastic fibers could have a mechanical role in the IVD. Elastic fibers were identified using arrows. The IVD, AF, NP, TZ, ILM, and PB represent the intervertebral disc, annulus fibrosus, nucleus pulposus, transition zone, interlamellar matrix, and partition boundaries (translemaller fibers), respectively. [Reproduced from references [41] subfigures (**a**–**e**); [42] subfigures (**f**,**g**); and [6] subfigure (**h**) with permission].

Within the NP, radially orientated elastic fibers appeared straight with more than 200 µm in length (Figure 3a). In addition, observing vertical or oblique elastic fibers in this region suggested that elastic fibers might run from endplate to endplate. They also reported a change in the orientation of elastic fibers at the interface of the NP and AF, creating a criss-cross pattern (Figure 3b), which seemed consistent with the report executed by Johnson et al. [38,41].

Within the AF region, a lower density of elastic fibers was observed in the lamella compared to the ILM and PB spaces (Figure 3c–e). The elastic fibers in the ILM and PB were reported to be randomly arranged and in parallel with collagen fibers in the lamella space [32,41], probably for recoiling the straightened collagen fibers. Similar structural features, including a higher density of elastic fibers between adjacent lamellae and long radially orientated elastic fibers in the NP, were found by Yu et al. (2005) in the human IVD [43]. Two studies reported sparse and disorganized elastic fibers in the AF lamellae of scoliotic compared to normal IVDs [43,44]. Akhtar et al. (2005) suggested the lamellar organization of collagen fibers was associated with elastic fibers present in the AF of normal human IVDs and that impaired regulation of collagen fibrillogenesis in the scoliotic IVDs might result in disruption of elastic fibers [45]. In 2006, Smith and Fazzarali identified the regional variation of elastic fibers (density and arrangement) in the AF of human IVDs using the resorcin-fuchsin technique [42]. They found different arrangements of elastic fibers between the lamella and interlamellar matrix, suggesting their multi-functional (structural and biomechanical) roles within the AF (Figure 3f,g). They also noted that the posterolateral region of the AF has a higher density of elastic fibers than the anterolateral region. For both regions, a higher density of elastic fibers was found for the outer than the inner regions of the AF [42]. Moreover, a detailed microstructural study to identify the distribution of elastic fibers, microfibrils, and collagen fibers in the human and bovine tail IVDs was performed by Yu et al. in 2007 [6]. They found a similar microfibrillar network for both species, with their organization varying across the IVD. They observed an abundant microfibrillar network that was highly colocalized with elastic fibers in the AF and oriented with the collagen fibers. The colocalization of microfibrils with elastic fibers in the inner AF was significantly lower than in the outer AF. They also found a similar crimped morphology for both microfibrils and collagen fibers in the AF lamellae, suggesting a noticeable structural relationship between these components. Moreover, few elastic fibers were detected within the NP of the bovine IVD. Their study revealed that elastic fibers were orientated almost parallel within the AF lamella of the human IVD and formed an angle to fibers in the adjacent lamella (Figure 3h) [6]. The function and structure of elastic fibers in the AF of a human IVD, as well as their plausible contribution to the IVD function, were discussed by Smith and Fazzarali (2009), suggesting the biomechanical role of elastic fibers in IVD function [46]. In 2015, Yu et al. carefully examined the elastic network in bovine IVDs, leading to a new interpretation of the AF structural organization [47]. They found that collagen compartments in the AF lamellae were enclosed and connected by an integrated network of elastic fibers. The mechanical interconnectivity between the AF elastic network and collagen compartments was examined qualitatively, revealing the contribution of elastic fibers to the structural integrity of two adjacent lamellae in the AF [47]. Table 2 shows the outcomes of studies (2002–2015) informing the organization and possible biomechanical function of elastic fibers in IVD.

**Figure 4:**
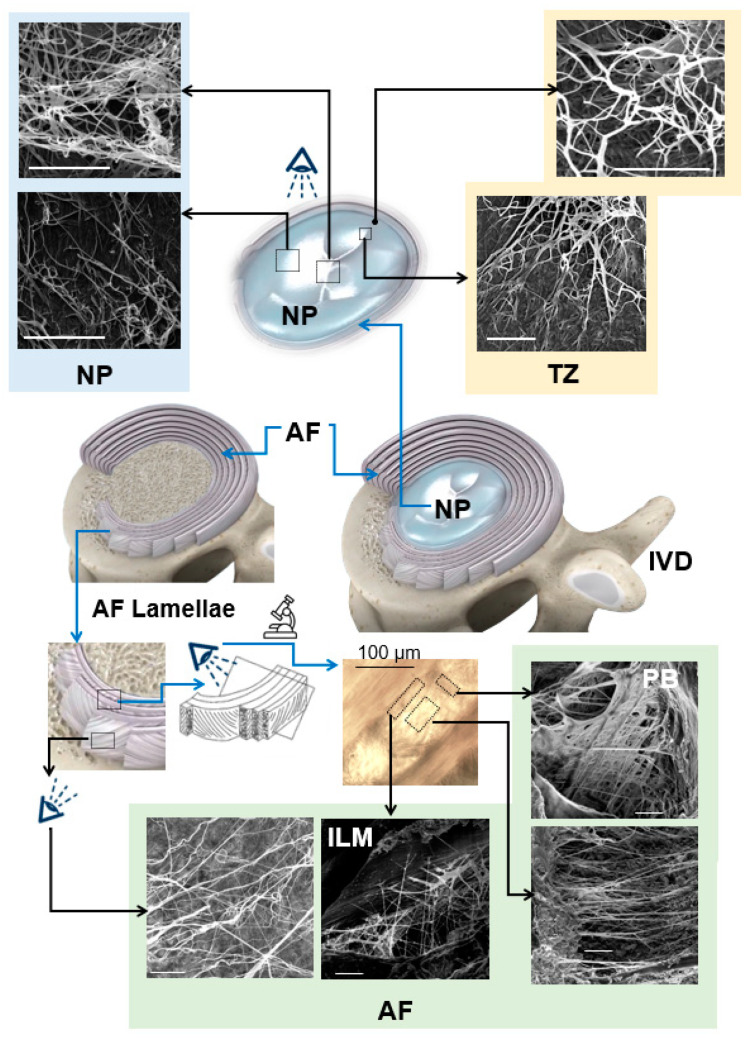
Scanning electron microscopy studies, based on alkali digestion with sonication, revealed the ultrastructural organization of elastic fibers in the IVD. Elastic fibers in the central NP are oriented in different directions. Radially orientated elastic fibers in the NP merge to create an elastic network at the TZ and extend to the AF, generating continuous and interconnected networks within the AF with higher density in the ILM and PB. This network is highly organized and represents an orthotropic structure. The IVD, AF, NP, TZ, ILM, and PB represent the intervertebral disc, annulus fibrosus, nucleus pulposus, transition zone, interlamellar matrix, and partition boundaries, respectively, and arrows (black and white) show the radial direction. Scale bars = 5 μm. [Reproduced from references [51] (NP); [52] (TZ); and [4,50] (AF) with permission].

A comprehensive review study by Tavakoli et al. (2016) explained the multi-scale hierarchical structure of the elastic fibers in the ILM and identified several unknown characteristics, including the ultrastructural organization of elastic fibers in the ILM, their mechanical properties, and their contribution to the AF structural integrity and delamination [3]. In 2017, a new alkali digestion technique was developed by Tavakoli et al. to remove all non-elastin components from the IVD, enabling in-situ visualization of elastic fibers of different species, including bovine, ovine, porcine, and human IVDs [48,49]. The utilization of this method revealed the ultrastructural organization of the elastic fibers in ovine IVDs for the first time at a nanoscale level (Figure 4). Their study identified a network of fine and thin elastic fibers that are interconnected by major, thick elastic fibers. Consistent with previous studies, they found a higher mass of elastic fibers in the ILM and PB compared to the lamella, while their distribution was approximately similar [4,50]. They revealed that elastic fibers create a fibrous network across the NP of ovine IVD, comprising thick (≈1 μm) and straight parallel fibers that were intertwined by fine (≈200 nm) wavy fibers. They identified that both straight (thick) and wavy (thin) fibers were frequently branched or merged to build a refined network of elastic fibers across the NP. They also observed entangled elastic fibers creating a high-density node in the NP that may contribute to its mechanical property [51]. In a recently published study, Tavakoli et al. (2020) utilized gradual elimination of non-elastin components from the interface of the AF and NP and showed that elastic fibers create a honeycomb structure in the transition zone [52,53]. This finding was consistent with other investigations that described a criss-cross arrangement of elastic fibers in the region [41]. They observed a delicate elastic network with lower density in the posterolateral compared to the anterolateral region of the transition zone [52].

Recent IVD microstructural studies using scanning electron and light microscopes have shown that elastic fibers are well organized across different regions of the IVD and generate a continuous and integrated elastic network. This network seems to play a critical role in the structural integrity and mechanical properties of the IVD as previously predicted, and therefore, its clinical role has to be identified in detail.

## 4. IVD Elastin Content

**Figure 5:**
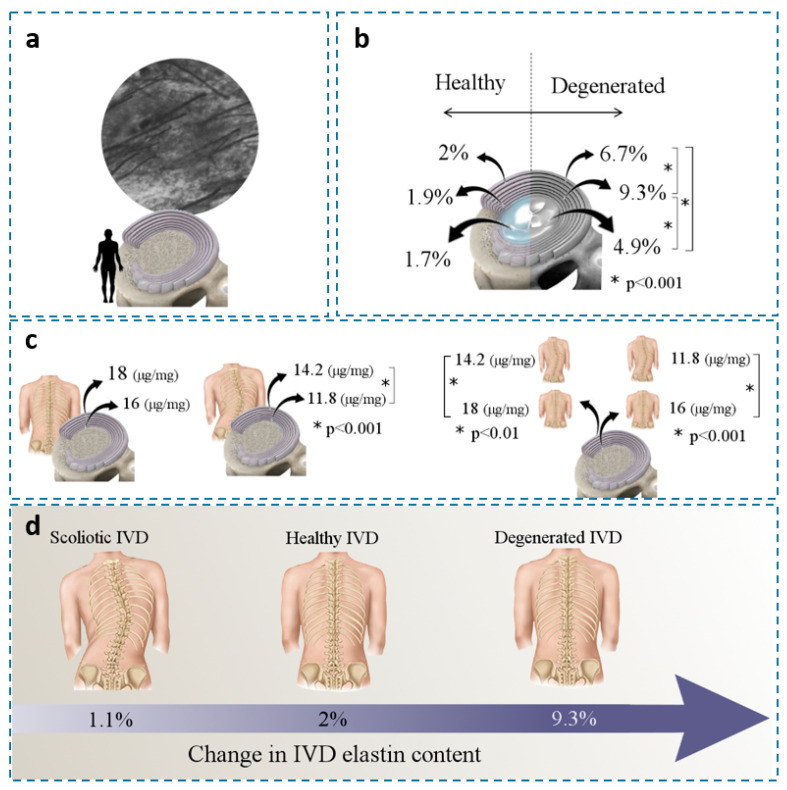
(**a**) A horizontal section of the AF (62-year-old male) indicates that elastic fibers occupy approximately 10% of the ECM in the AF (×2000) [39]. (**b**) Changes in elastin/IVD dry weight in healthy and degenerated IVDs at different locations; mean values were presented [54]. (**c**) Change in elastin/dry mass (μg/mg) in the AFs of healthy and idiopathic scoliotic IVDs [55] and (**d**) Change in elastin content for scoliotic, healthy, and degenerated IVDs (dry weight term). [Figure 1a reproduced from reference [39] with permission].

In 1985, Johnson et al. utilized light microscopic and histological techniques to quantify the relative number of elastic fibers (percentage) in the AF of human IVDs (Figure 5a) and estimated that 10% of the AF area was occupied by elastic fibers [39]. Mikawa (1986) found no significant difference in elastic fiber content between the NP and AF, with the overall elastic content being 1.7% of the IVD dry weight [40]. In 2007, Cloyd and Elliott reported a similar elastin content for the NP, inner, and outer AF of healthy human IVDs, where approximately 2% of the IVD’s dry weight was elastin [54].

Studies have shown that elastin content correlates with disc degeneration and other diseases. Cloyed and Elliott (2007) found a significant increase in IVD elastin content (dry weight term) for degenerated (grades > 2.5) compared to healthy IVDs. The largest amount of elastin was found in the inner AF (9.3%) compared to the outer AF and the NP (Figure 5b) [54]. Since elastin content was normalized to collagen content, the higher elastin density in the inner AF might correlate with the mechanical loads to restore AF integrity under radial loading. Kobielarz et al. (2016) used an enzyme-linked immunosorbent assay (ELISA) to quantify elastin content in the AF of thoracolumbar and lumbar scoliotic IVDs collected from young patients (<20 years old). Compared to the healthy IVDs, they showed a lower elastin content for scoliotic IVDs for both inner and outer AF. The elastin content for the outer and inner AF of healthy IVDs was 18 and 16 µg/mg (dry weight terms), respectively. Whereas the scoliotic IVDs decreased to 14 µg/mg (outer AF) and 11 µg/mg (Figure 5c) [55]. These studies indicated a strong positive correlation between elastin content and IVD degeneration (Figure 5d).

## 5. Development and Aging

**Figure 6:**
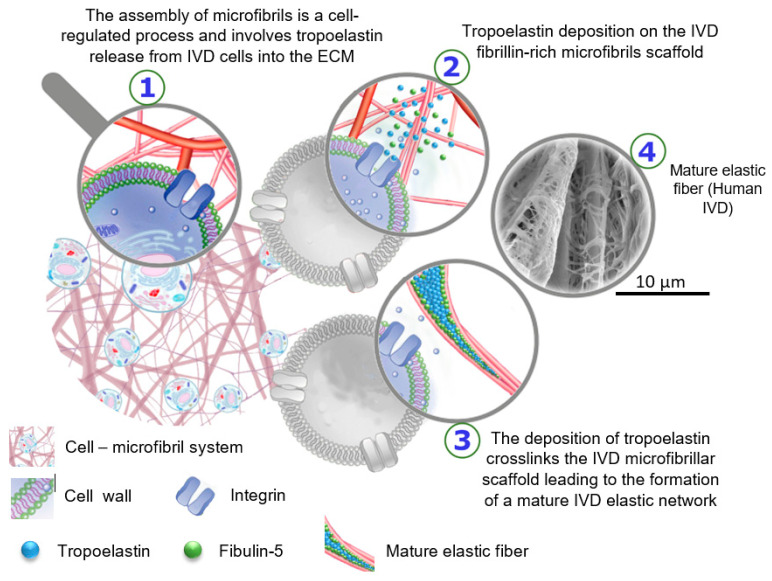
Schematic drawing to represent the biological development of elastic fibers in the intervertebral disc. The SEM image was prepared using alkali digestion with sonication technique to isolate elastic fibers in situ displays a mature elastic fiber in a human IVD, which consists of a core of amorphous elastin surrounded by microfibrils [The SEM image from Tavakoli’s research lab].

The biological development of elastic fibers in the IVD is not very well known; however, general studies have shown that the early development of elastic fibers involves tropoelastin deposition on a fibrillin-rich microfibril scaffold [56]. Similar to other ECM macromolecules, the assembly of microfibrils is a cell-regulated process and involves tropoelastin release from cells into the extracellular space. The deposition of tropoelastin crosslinks the microfibrillar scaffold, leading to the formation of a mature elastic network (Figure 6) [57]. In the early development stage, elastic fibers are surrounded by a large portion of microfibrils; however, their concentration progressively decreases during maturation, leading to central elastin formation. Mature elastic fibers are comprised of a core elastin protein enclosed by a microfibrillar mesh with some proteoglycans, i.e., versican, decorin, and biglycan, taking critical parts in the integration of the mesh into the adjoining ECM [58,59,60]. At the interface of elastin and microfibrils, the ECM glycoprotein emilin (elastin microfibril interface located protein) and fibulin-5 are found [61]. Emilin and fibulin-5 are deemed responsible for the deposition of tropoelastin onto the microfibrils and linking the elastic network to cells, respectively.

Melrose et al. (2011) reported colocalization and interaction between elastin and perlecan during elastic network assembly, indicating the important role of elastin in the development of AF in human fetal and newborn ovine IVDs [62]. Elastic fibers were observed by Buckwalter (1998) to lie parallel to the collagen fibrils and create a sheet in the AF and the NP of newborns [63]. A study to understand the morphologic changes during aging was performed by Postacchini et al. in 1984 using rat IVDs (newborns, young, and old). They showed that elastic fibers are present in both immature and mature IVDs (AF region); however, their density was lower in the mature IVDs than in the young ones [64]. In 1985, Johnson et al. indicated a gradual decrease in the number of elastic fibers by age, with a significant difference observed from the third to the seventh decade. Slight changes were reported in the number of elastic fibers from one to the next decade [39]. Olczyk (1994) reported a systematic increase in elastin relative to the glycosaminoglycan during the first four decades of life, with the ratio remaining constant in later life [65]. In 2006, Smith and Fazzarali measured the density of elastic fibers in different regions of the AF in human lumbar IVDs using a small sample size (7 IVDs). They used an image processing technique on histologically prepared AF samples and found a higher density of elastic fibers in the posterolateral compared to the anterolateral region of the AF. Without statistical validation, an increasing trend for elastic fibers between the ages of 16 to 40 was reported [42]. Melrose et al. (2007) studied the changes in the number and composition of trans-lamellar cross-bridges in the AF of ovine IVDs [66]. Cross-bridges, also known as PBs, were located between collagen bundles that transverse and interconnect several lamellae in the AF [4,47,67]. They showed that aggrecan and versican were localized in the cross-bridges with their quantity increased during aging. They also reported a higher number of cross-bridges in the anterior compared to the posterior region of the adult IVDs. They observed type IV collagen, which was specifically confined to the cross-bridges region, with a higher concentration found in the adult (6-year-old) compared to the younger (2-week-old) IVDs. The structural changes that occurred with skeletal maturity might have offered a functional adaptation to the loads experienced by the AF [66]. Meanwhile, the research conducted by Melrose et al. did not investigate the presence of elastic fibers in the translamellar cross-bridges, but a delicate and organized network of elastic fibers was observed in this region, indicating the change in elastic fiber organization during aging [4,6,43]. In 2012, Siva et al. measured the accumulation of aspartic acid and pentosidine biomarkers to study the longevity of elastin in cervical human IVDs [68]. Their results showed that elastin was metabolically stable for both healthy and degenerated IVDs, with the sign of new elastin synthesis in degenerated IVDs above the mid-50s [68]. In 2019, Fontes et al. used human L5-S1 IVDs to explore the relationship between aging and elastic fibers within two groups of young people (<35 years) and the elderly (>65 years). They found abundant elastic fibers in young people’s IVDs compared to the ones of the elderly and concluded that the presence of elastic fibers is correlated more to age than degeneration grade [69].

## 6. IVD Elastic Fibers and Cells

Studies have shown that fibulin-5 protein regulates elastic fiber organization and plays a role in binding cells to elastic fibers through colocalization with elastic microfibrils and interaction with integrin cell receptors [70,71]. Using 14-week-old human IVDs, fibrillin-1 fibrils were also observed attached to outer AF cells [62]. Elastin in the AF of newborn ovine IVDs was also observed near the pericellular matrix components, mainly perlecan, which suggested perlecan’s role in depositing elastin into the ECM [62]. Perlecan was found to stabilize growth factors and present them to the cell receptors, leading to their activation, therefore playing a vital role in initiating cell signaling [72]. In addition, another study identified the contribution of perlecan in fibrillin-1 assembly or its deposition in human fetal IVD (14-week-old) and visualized fibrillin fibrils providing a cell-ECM connection. This might suggest that fibrillin facilitates communication between IVD cells and their microenvironment [73]. The interaction between perlecan with fibrillin-1 and tropoelastin, occurring during microfibril development, along with fibrillin-1 regulating growth factors’ bioavailability, suggests a role for the elastic network in mechanotransductive processes. Apparently, this interaction leads to the creation of links between cells and cell-ECM, resulting in IVD cells contributing to mechanosensory processes essential for ECM remodeling and IVD tissue homeostasis [62,74,75,76].

The relationship between the structure of IVD elastic fibers and cell morphology is not very well understood. Few studies have identified different cell morphologies in the ILM compared to the lamellae. Cord-like, spherical, and flattened cells were observed in the ILM region of the AF, moving from the outer towards the inner AF, respectively, with the ILM’s cells creating a connective network in the outer AF. The ILM’s cells rarely form a network in the inner AF [77,78,79]. The density of elastic fibers is high in the ILM, and the ILM biochemical properties are different compared to the lamella. Therefore, it is considered that the ILM cells might have different phenotypes compared to the cells in the AF lamellae [78]. While the general belief is that the regional variations in the ILM’s cell morphology might reflect different mechanical loading experienced by the AF, the impact of elastic fiber organization on cell morphology is not yet clear.

## 7. Function and Mechanical Properties of IVD Elastic Fibers

**Figure 7:**
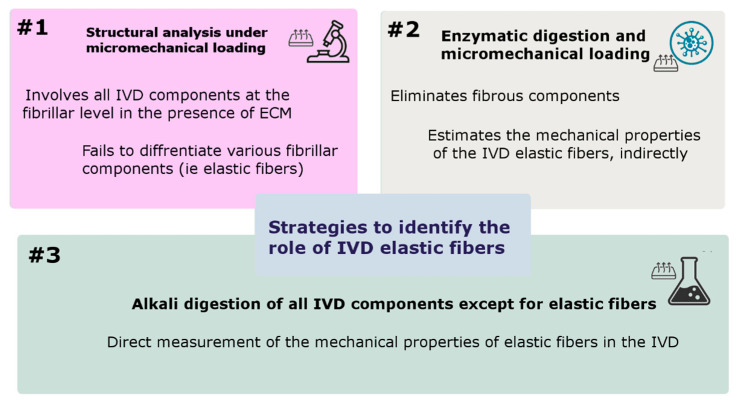
Most frequently used strategies to identify the function of IVD elastic fibers and measure their mechanical properties.

Early IVD structural studies at the micro-level have identified low density with a random distribution of elastic fibers and therefore thought that the role of elastic fibers was not substantial in the overall IVD mechanical properties. However, biochemical research has revealed a higher concentration of polar amino acids in elastin extracted from IVD than in ligamentum nuchal, suggesting a strong crosslink between elastic fibers and extracellular components; hence, their contribution to the IVD mechanical function. In addition, recent studies have found a highly organized elastic network across the IVD with different regional distributions. The presence and colocalization of Versican with the elastic network in the ILM, reported by Melrose et al. (2001), suggested an anchoring role of elastic fibers connecting adjacent AF lamellae [80]. Moreover, it was thought that the dense and randomly organized elastic network in the ILM might facilitate relative sliding of adjacent lamellae, by which the elastic network contributes to the recovery of the AF lamellae after deformation [81,82]. With such observations, it was believed that elastic fibers played a role in the structural integrity of IVD and contributed to its recovery after loading. To understand the mechanical role of elastic fibers in IVD, three different approaches have been suggested (Figure 7).

### 7.1. Structural Analysis under Micromechanical Loading

The first approach has been the evaluation of structural connectivity between IVD components at the fibrillar level in the presence of ECM (Figure 7) [5,47]. This approach has often been utilized in combination with light microscopy, histology, and micromechanical testing to investigate the structural mechanisms that create integration between the IVD anatomical regions. Unfortunately, this approach has failed to differentiate various fibrillar components (collagen, elastin, etc.) and has not been able to address the mechanical role of elastic fibers. However, researchers have combined the results from this approach with the regional distribution of elastic fibers to speculate on how elastic fibers might contribute to the structural integrity of IVD. Of particular interest, efforts to measure the mechanical properties of the ILM may reflect the contribution of elastic fibers to the structural integrity of the IVD since elastic fibers are the main component of the ILM. Iatridis and Gwynn (2004) reported an increase in the ILM shear stress with IVD degeneration, indicating that the mechanical properties of elastic fibers in the ILM were likely associated with the propagation of circumferential tears in the AF often observed in degenerated IVDs [81]. In 2006, Pezowicz et al. reported that the interlamellar matrix, consisting of a complex fibrous structure, provided structural cohesion between the collagen fibers of two adjacent lamellae in bovine (oxtail) IVDs [83]. They also found a complex set of interconnecting fibrous structures transverse to the collagen direction that contributed to the structural integrity of the AF lamella with a peak stress of 0.3 MPa at a 2.2 stretch ratio [84], which is compatible with the mechanical properties of elastin [31]. Subconsciously, important research has been done that has indirectly identified the mechanical and functional contribution of elastic fibers in the IVD. Different approaches, including AF delamination, tears, patterns of IVD herniation, and lamellae peel strength, have suggested the plausible role that elastic fibers play in providing AF structural integrity [85,86,87,88]. One study revealed a higher stiffness (under radial loading) for the lamella interface (ILM) compared to the corresponding lamella values, with higher ILM stiffness for the outer AF compared to the inner AF. This finding might reflect the contribution of the ILM’s elastic fibers to the structural integrity of the AF [89]. Schollum et al. (2008) used ovine IVDs to evaluate the role of elastic fibers in the structural integrity of the AF and observed a high level of connectivity between the ILM and PB. They observed infrequent substantial radial connections spanning several lamellae, with their degradation anticipated to link with AF weakening and IVD mechanical failure [5]. Veres et al. (2008) and Pezowicz (2006) also found a weak ILM cohesion for the outer posterior AF lamellae under high NP pressure that might indicate the contribution of elastic fibers in the herniation process [90,91]. However, pressurizing the NP to identify the AF failure pathway might not be clinically relevant as a set of disruptive events was sought and considered for weakening both the AF lamellae and ILM [91]. Wade et al. (2011–2012) investigated the structural integration across an ovine NP and endplate junction and proposed that the NP fibrils penetrated the endplate and were closely packed with the endplate multidirectional fibrillar structure [92]. While the nature of the fibers (elastic or collagen) was not identified in their study, Wade et al. found a structural continuity across the NP (from vertebra to vertebra of ovine IVD) with the NP-endplate interface supporting an average tensile force of 20N before failure [93]. Furthermore, they found structural connectivity between the AF and NP of ovine IVDs, imposed by a highly organized fibrillar network, supporting an average tensile force of 5.7 N in the radial direction [94]. Yu et al. (2015) identified a well-organized network of elastic fibers in the AF of bovine IVDs that appeared to provide a mechanical linkage across the AF and maintain the AF integrity during the radial stretch. This study involved chemically fixed stretched AF samples that were analyzed optically using immunohistology, and therefore, the structural distortion of both elastic fibers and microfibrils was observed in the stretched AF [47]. Vergari et al. (2016) used second harmonic generation microscopy and found a higher linear strain (3 times) for the ILM compared to the lamellae in the outer AF of bovine IVD. However, no slippage at the ILM junction was found, confirming that the AF strain was mainly due to collagen bundle rotation rather than elastic network alteration [95]. The viscoelastic and failure properties of the ILM in both the tension and shear direction of loading were measured by Tavakoli and Costi (2018) using ovine IVDs (Table 3). This study revealed a significantly higher stiffness and lower energy absorption for the ILM at faster compared to the slower strain rates. However, the failure properties of the ILM (strain rate = 10%) were not significantly different under tension and shear loadings [96].

Furthermore, Tavakoli et al. (2018) performed a multiscale biomechanical and structural study to understand the role of the ILM elastic fibers during progression to herniation and suggested that the ILM biomechanical properties (such as modulus and maximum stress) were significantly reduced compared to the lamella during progression to herniation. Their study confirmed that the ILM, compared to the AF lamella, was the weaker AF component during the progression of herniation in the posterolateral region of the IVD. This study proposed a stress threshold above which the ILM fails mechanically [97]. While these studies have enhanced our knowledge about the structural integrity between IVD components at the fibrillar scale, they were incapable of differentiating between collagen and elastic constituents, thus, the specific role of elastic fibers remains unclear. It is important to note that a new study to quantify the deformation of intact IVD under compression using synchrotron X-ray micro-tomography has revealed the impact of the IVD microstructure on associated 3D strain patterns and reported a similar distribution for strain patterns compared to the microstructural organization of elastic fibers [98].

### 7.2. Elastin Enzymatic Digestion and Micromechanical Loading

Enzymatic treatment to eliminate elastic fibers has been the second most frequently used strategy for measuring the IVD mechanical properties in the absence of elastic fibers (Figure 7). This strategy is relatively expensive, time-consuming, suffers non-specific degradation of other ECM components, and estimates the mechanical properties of the IVD elastic fibers indirectly. In 2008, Smith et al. investigated the contribution of elastic fibers to the tensile mechanical properties of the AF using human IVD. This study found a significant reduction in toe (from 70 to 4 kPa) and linear (from 210 to 20 kPa) moduli, as well as a significant increase in extensibility (from 16% to 93%) of the AF after the elimination of elastic fibers. The scale of this contribution was remarkable, as elastin content in the AF is relatively small [99]. Michalek et al. (2009) measured the AF micro-deformation of non-digested and elastase-treated bovine IVDs under dynamic shear loading and tracked the stretch and rotation of collagen fiber bundles. Their study revealed that lamellae sliding did not occur at the ILM junction, and the mechanical properties of the ILM were mostly controlled by collagen fibers and fibrillin rather than elastic fibers [100]. Isaacs et al. (2014) used human IVDs to examine the impact of elastin and collagen digestion on the elastic and failure properties of the IVD. They found a significant reduction in the mechanical properties of the ILM and lamella in the digested samples. Their study also revealed that the change in failure strain for samples that were digested by elastase was not significant compared to undigested samples, suggesting that collagen fibers played an important role in the IVD failure strain [101].

### 7.3. ECM Alkali Digestion and Micromechanical Loading

Digestion of all IVD components except for elastic fibers has been the third strategy that provided an opportunity for direct measurement of the mechanical properties of elastic fibers in the IVD (Figure 7). One study utilized this strategy (Tavakoli and Costi, 2018, [96]) and provided new insight into the viscoelastic and failure mechanical properties of the ILM elastic fibers (Table 3). They found that the ILM elastic fibers exhibited a significantly higher capacity for energy absorption at slow compared to medium and fast strain rates. This study also revealed a higher tensile failure force for the ILM elastic network compared to the shear direction, which was consistent with the orthotropic structural organization of the ILM elastic fibers [102].

This strategy was successfully applied to the ILM and has a great potential to directly measure the mechanical properties of the elastic fibers in different regions of the IVD. It is expected that the third strategy will be used for future investigations to understand the relationship between IVD disease (i.e., degeneration) and the mechanical properties of elastic fibers. Table 4 shows diverse approaches to identifying the contribution of elastic fibers in the IVD using three different strategies and the relevant key findings.

## 8. Future Outlook

Understanding the structure–function relationship of the IVD components, at different scales from micro (collagen and elastic fibers) to macro (NP, AF, and TZ), plays an important role in developing new tissue engineering strategies [103,104,105]. Of particular importance, the differences in the biomechanical properties of the IVD are likely to result predominantly from the IVD structural organization rather than compositional variation [106]. Structural and biomechanical studies have shown that elastic fibers contribute significantly to the structure and function of IVDs; however, more studies using advanced technologies are required to fully characterize their mechanical properties, including in health and disease conditions [107,108,109,110]. While recent studies have identified that elastic fiber breakdown products, such as elastokines, play a role in the development of a variety of diseases, i.e., supravalvular aortic stenosis and the Williams-Beuren syndrome, the impact of the process on IVD pathologies is yet unclear [111,112]. It seems that the future direction in IVD matrix biology can include studies to enhance our understanding of the biology of the processes of IVD elastogenesis and elastic fiber decay. In this regard, performing IVD proteomic studies offers quantitative analysis of complex protein mixtures in healthy, degenerated, and aged IVDs. The study of protein solubility, profile, extractability, and expression with age and in healthy and degenerated conditions using proteomics can identify relevant similarities and differences in ECM between healthy and degenerated IVDs of different ages. It is expected that advanced proteomic methods can enhance our understanding of IVD elastic fiber synthesis and degradation, leading to the identification of their role in IVD biology [113,114,115]. This may contribute to the development of innovative strategies and advanced biomedical materials for IVD regeneration or the treatment of age-related conditions [116,117,118]. From an engineering point of view, the focus of future research should be the creation of more realistic IVD models. Incorporating the role and organization of elastic fibers in the design and fabrication of IVD tissue-engineered scaffolds and computational modeling are two important future directions.

### 8.1. Elastic Fibers and Full IVD Scaffold Design

Regeneration of IVD requires deep knowledge about mechanisms of IVD degeneration and is a challenge at the intersection of IVD biology, biomaterials, and biomechanics which would benefit from having a reproducible and adaptable 3D scaffold able to recapitulate the relevant complexity of the IVD [119,120,121,122,123,124]. The available IVD scaffolds are standard in vitro cultures in 2D and 3D, ex vivo systems employing human or animal IVDs and bioreactors [125,126]. A 2D cell culture is obviously an oversimplified approach. Cultures with cell encapsulation provide a 3D environment, however, they fail to capture the gradients of composition and the structural organization, which are essential for mimicking IVD. For example, there are different ways cells sense the stress distribution within a 3D gel in a cell culture dish or within an IVD. The construction of a whole IVD has been the focus of a limited number of studies, and often three different fabrication strategies have been used. The most common strategy has been the assembly of individually prepared cell-seeded AF and NP [127,128,129]. The most advanced IVD-like construct consists of a circular fibrous structure with or without angle-ply architecture using electrospinning or fiber deposition and then the addition of a hydrogel at the core of the ring for the NP [130,131,132,133,134]. The electrospinning approach is capable of developing a lamellar structure to resemble the organization of native AF, which is important for modeling the mechanical properties and formation of oriented ECM. However, these engineered IVDs lacked the gradient composition, integration between the AF and NP, and elastic fibers’ structural organization [135,136,137].

The fabrication of integrated biphasic AF-NP scaffolds has been the second strategy to develop full IVD scaffolds. Researchers have often employed three approaches, including 1—chemical routes to integrate the AF and NP during fabrication, 2—acellular matrix-derived biomaterials, or 3—naturally aligned plant-based platforms, to create a fully integrated IVD scaffold [138,139,140]. This strategy was able to mimic the structural organization of the IVD at the macroscale level, with the AF-NP integration being another advantage in enhancing the overall mechanical properties of the scaffold. However, this approach cannot simulate the microstructure of the IVD, thus the function and organization of elastic fibers have been overlooked [141,142,143,144,145].

The third strategy has been the cellularization of acellular animal IVDs [146,147,148]. Considering the limitations of other strategies, ex vivo models are better options, especially in combination with bioreactors. However, using human or animal tissues imposes a strong bias depending on the donor. Human donors are scarcely available, and each one comes with a specific set of co-morbidities, genetic background, degeneration state, traumatic lesions, and an unknown lifestyle. Animal IVDs, for example, from the ovine spine or bovine tails, are commonly used; however, they are different from human IVDs, as these animals do not stand on two legs, and tails are only loaded from muscle forces. Consequently, with a substantial change in spine biomechanics, the tissue is often harvested from young animals, and its composition, developmental origin, and biology are different from humans [149]. Similarly, preclinical studies with such scaffolds have severe limitations regarding their recapitulation of the human IVD in size, mechanics, and biology; research ethics and guidelines underline the need to reduce, refine, and replace animals in research [150,151].

The application of additive manufacturing in the fabrication of full IVD scaffolds will provide opportunities for creating more realistic IVD scaffolds with defined regions and gradient areas between AF and NP, replicating the composition and structural organization of the native IVD [152,153]. It is expected that IVD scaffolds produced by biofabrication techniques enable the change of single parameters independently of all the others. For example, it would allow embedding of juvenile cells in IVD models with mechanical properties matching older or degenerative tissues, or cells from old donors in young-like IVDs, facilitating targeted investigations into the finely regulated interplay between mechanics and cell biology.

### 8.2. Refined Computational Modeling

Finite element models have significantly contributed to our understanding of IVD functional biomechanics. They have played an essential role in minimizing the use of animal and human IVD models in experimental biomechanics and pre-clinical studies. Various assumptions and degrees of complexity have been employed to model IVD or its components (mainly the AF and NP), where IVD was considered a homogeneous, heterogeneous, or fiber-reinforced composite entity [19,154,155,156,157,158,159,160]. Unfortunately, the role of elastic fibers and their contribution to IVD mechanical behavior under complex physiological loading modes have not been studied yet. A refined representation of the elastic fibers in the IVD would shed light on their mechanical role in the IVD.

Furthermore, the refined representation of fibers, including the contribution of fiber-matrix interaction (structure-based finite element modeling) in computational models, demonstrated superior results to homogenized models [161]. These models usually consist of a refined representation of the angle-plied circumferential collagen fibers in the AF and separate between the material properties of the fibers and ground substance [127,162,163,164,165].

Few computational studies have considered the mechanical role of the ILM using homogenized IVD models that include the ILM region in the studies. In 2011, Nerurkar et al. considered a hyperelastic model for interlamellar interactions in angle-ply biologic laminates based on a tissue-engineered construct to understand how the ply angle changed with uniaxial extension. They found a reinforcing role of interlamellar shearing and characterized the contributions of extrafibrillar matrix, fibers, and interlamellar interactions [166]. Labus et al. (2014) developed a hyperelastic constitutive computational model to describe the local shear stress–stretch relationship for the lamellae and across the ILM of the AF and found a higher shear modulus for the lamellae compared to the ILM [167]. Adam et al. (2015) utilized an image-based model and came to the understanding that the ILM shear resistance conferred the IVD compressive stiffness [168]. Mengoni et al. (2015) developed a finite element model to assess the mechanical behavior of the ILM in the AF and derived the interface stiffness values for the outer and inner AF. They found that the interface stiffness was higher (40–70%) for the outer AF compared to that of the inner AF [89]. In 2019, Kandil et al. developed a microstructure-based chemo-viscoelastic homogenized model to evaluate the ILM-induced time-dependent response to loading. Their results revealed that the ILM time-dependency response could be explained based on the combination of the ECM intrinsic viscosity and the internal fluid transfer [169]. All of the above considered the ILM as a homogenous entity without a refined representation of the fibers. Recently, Sharabi et al. (2019) performed a refined finite element study to understand the mechanical role of the translamellar radial fiber network in the AF and observed that the local stress and strain were decreased (by 10% and 25%, respectively) in the presence of the radial fibers, together with creating an entangled network of fibers with the collagenous circumferential fibers. This indicated that the fiber network plays a crucial role in reducing the stresses and strains in the AF lamellae under lateral bending, flexion, and extension modes [162]. Recently, Ghezelbash et al. (2021) demonstrated a nonhomogenous full IVD model that consists of the circumferential collagen AF, ILM, as well as elastic fibers and compared their results to experimental results [155].

To the best of the authors’ knowledge, a full IVD computational model to represent the contribution of the elastic network to the mechanical properties and structural integrity of the IVD is still lacking. Since our understanding of how elastic fibers are organized in the different regions of the IVD has been enhanced and several studies have identified the mechanical properties of the IVD elastic fibers, the development of more physiologically relevant computational IVD models is expected.

## 9. Conclusions

The purpose of this literature review was to address the current knowledge about the role of elastic fibers in the intervertebral disc. During the early studies, the function and the structural organization of elastic fibers in soft tissues such as lungs, blood vessels, and skin were examined in detail, whereas their characteristics in the IVD did not get enough attention. However, recent studies demonstrate that elastic fibers play a crucial role in the mechanical function of IVD. These new studies have given us a new understanding of the multi-scale hierarchical structure of the elastic fibers and their mechanical role in the structural integrity of the IVD. Recently, there have been a few attempts to develop IVD scaffold designs using different fabrication techniques. Unfortunately, these IVD models often lacked the ability to display gradient composition and the structural organization of elastic fibers. Future IVD models should represent the role and structural organization of elastic fibers to represent more physiologically relevant models.

On the other hand, the mechanical contribution of elastic fibers has been ignored inevitably in most computational models, as the role of elastic fibers in the IVD was not well understood. As our understanding of the characteristics of elastic fibers has enhanced over time, better multi-scale computational models can be introduced. From a clinical point of view, future studies should aim at identifying the role of elastic fibers in the pathophysiology of lower back pain.

## Figures and Tables

**Table 1:** Early attempts to identify the presence and arrangement of elastic fibers in the IVD.

Sample	Key Finding	Method	Ref.
Human IVD	Elastic fibers were found in the human IVD for the first time.	Transmission electron microscopy	[1]
Human IVD	The presence of elastic fibers at the AF and NP interface was reported.	Stained thin tissue (uranyl acetate and lead citrate) and transmission electron microscopy	[2]
Human fetal AF	Elastic fibers were found to be immature, consisting of a bundle of microfibrils with 20 nm in diameter.	Stained thin tissue (uranyl acetate and phosphotungstic acid) and electron microscopy	[3]
Human cervical IVD	The presence of a three-dimensional lattice of elastic fibers at the NP-endplate interface was reported.	Stained thin tissue (orcein, hematoxylin, and eosin) and transmission electron microscopy	[4]
Dog IVD	Elastic fibers were found in both the intra- and inter-lamellar regions of the AF.	Stained thin tissue (orcein, hematoxylin, and eosin) and transmission electron microscopy	[5]
Human lumbar IVD	Elastic fibers were found to occupy approximately 10% of the extracellular matrix of the AF (1985).	Stained thin tissue (orcein, hematoxylin, and eosin) and light microscopy	[6]
Human IVD	Elastin content was found to be less than 2% of the total IVD dry weight and elastic fiber in the intervertebral disk ere very sparse and irregular.	Immunohistochemistry and light microscopy for structural analysis and hot alkali method for elastin content measurement	[7]

**Table 2:** Studies that reported organized distributions of elastic fibers in different regions of IVD.

Sample	Key Finding	Method	Ref.
Bovine IVD	Abundant and organized distribution of elastic fibers in different regions of IVD.	Stained thin tissue (orcein and immunostaining) and light microscopy	[8]
Human IVD	High-density and long radially oriented elastic fibers in the ILM and NP, respectively.	Stained thin tissue (orcein and immunostaining) and polarized light microscopy	[9]
Human lumbar IVD	Disorganized elastic fibers in the AF lamellae of scoliotic compared to normal IVDs.	Stained thin tissue (orcein and immunostaining) and light microscopy	[9,10]
Human lumbar IVD	Regional variation of elastic fibers (density and arrangement) in the AF.	Stained thin tissue (resorcin-fuchsin) and light microscopy	[11]
Human and bovine IVD	In the AF, the alignment of microfibrils and elastin fibres were similar to that of the collagen fibres. In the NP, microfibrils were mostly arranged around the cells and elastin fibers were rarely detected.	Stained thin tissue (dual immunostaining) and light microscopy	[12]
Bovine caudal IVD	Collagen compartments in the AF lamellae are enclosed by an integrated network of elastic fibers.	Immunohistology and confocal microscopy using unloaded or radially stretched tissues	[13]

**Table 3:** Mechanical properties of the ILM and its elastic fiber network (ovine IVD) [96,102] reported in the radial (R) and circumferential (C) directions of loading. Phase angle represents the hysteresis curve (energy absorption) between the loading and unloading cycles. Extensibility refers to the strain at the intersection of the toe and the linear stiffness of the loading cycle. Failure stress and strain are defined as the peak stress and the corresponding strain, respectively.

Mechanical Properties	Strain Rate (%s^−1^)	Load Direction	ILM	ILM Elastic Network
Phase angle (°)	0.1	R	13.5 ± 3.6	6.5 ± 3.4
		C	12.5 ± 2.9	15.3 ± 6.7
	1	R	11.5 ± 3.1	6 ± 2.3
		C	8.9 ± 2.7	10.5 ± 3.6
	10	R	5.1 ± 3.2	1.6 ± 0.9
		C	2.5 ± 1.2	2.1 ± 0.8
Extensibility (%)	0.1	R	26.2 ± 6.3	30.5 ± 20.1
		C	30.4 ± 11	31.6 ± 10
	1	R	27.6 ± 4.2	27.7 ± 16
		C	22.4 ± 4.6	25.3± 12
	10	R	27.4 ± 6.5	19.4 ± 9
		C	21.8 ± 5.1	20.4 ± 8.9
Modulus (kPa)	0.1	R	1.3 ± 0.5	0.8 ± 0.3
		C	1.0 ± 0.4	0.5 ± 0.3
	1	R	1.8 ± 0.6	0.8 ± 0.4
		C	1.1 ± 0.3	0.5 ± 0.3
	10	R	1.8 ± 0.5	0.6 ± 0.3
		C	1.2 ± 0.5	0.6 ± 0.2
Failure stress (kPa)	10	R	287 ± 58	225 ±66
		C	302 ± 46	149 ± 47
Failure strain (%)	10	R	256 ± 80	308 ± 78
		C	343 ± 154	473 ± 256

**Table 4:** Key findings relevant to the mechanical function of IVD elastic fibers.

Sample	Key Finding	Ref.
Strategy 1—Structural analysis in the presence of ECM under micromechanical loading		
Rat tail	Mechanical properties of elastic fibers in the ILM were likely associated with the propagation of circumferential tears in the AF	[14]
Ox tail	The ILM provides structural connectivity between the AF layers	[15]
	Elastic fibers may contribute to the structural integrity of the AF lamella with a peak stress of 0.3 MPa	[16]
Human	The annular delamination strength of the AF was measured	[17]
Rabbit	IVD degeneration affects the ILM (elastic fibers) and reduces the AF deamination strength.	[18]
Ovine	A high level of connectivity between the ILM and PB was found in the AF	[19]
	A higher tensile stiffness was found for the ILM compared to that of the AF lamella (the contribution of the ILM’s elastic fibers to the structural integrity of the AF)	[20]
	The ILM exhibited weak connectivity in the posterior region of the AF, creating a pathway for IVD herniation while pressurizing the NP	[21,22]
	The NP microfibrils penetrate the endplate	[23]
	NP-endplate interface supporting an average tensile force of 20 N before failure	[24]
	The AF-NP interface tensile force in the radial direction was measured (7.7 N)	[25]
	The ILM was fully characterized mechanically in both shear and tension directions of loading	[26]
	The ILM mechanical properties, compared to the lamella, were significantly reduced during progression to herniation.	[27]
Bovine	A well-organized network of elastic fibers to provide a mechanical linkage across the AF	[13]
	A higher linear strain (3 times) for the ILM was found compared to the lamellae	[28]
Strategy 2—Elastin enzymatic digestion and micromechanical loading		
Human	The elimination of elastic fibers from the AF was shown to decrease modulus while increasing the AF extensibility.	[29]
	The impact of elastin and collagen digestion on elastic and failure properties of the IVD was examined	[30]
Bovine	The mechanical properties of the ILM were mostly controlled by collagen fibers and fibrillin rather than elastic fibers	[31]
Strategy 3—ECM alkali digestion and micromechanical loading		
Ovine	The viscoelastic and failure mechanical properties of elastic fibers in the ILM were characterized.	[32]

## Data Availability

Not applicable.
